# Comparative study of the functional outcomes of combined medial-lateral approach arthrolysis with or without external fixation for severe elbow stiffness

**DOI:** 10.1186/s12891-021-04796-3

**Published:** 2021-11-10

**Authors:** Dan Xiao, Maoqi Gong, Chen Chen, Yejun Zha, Ting Li, Shangwei Ji, Kehan Hua, Weitong Sun, Xieyuan Jiang

**Affiliations:** Department of Orthopedic Trauma, Beijing Jishuitan Hospital, No.31 Xinjiekou East Street, Xicheng District, Beijing, 100035 China

**Keywords:** Elbow, Stiffness, Combined medial-lateral approach, Arthrolysis

## Abstract

**Background:**

To evaluate and compare the functional outcomes of combined medial-lateral approach open arthrolysis with and without hinged external fixation.

**Methods:**

We retrospectively collected and analyzed the clinical data of patients with severe elbow stiffness who were treated in our institution from January 2018 to January 2019. All of them were treated with combined medial-lateral approach arthrolysis. There were 20 patients who had the hinged external fixation placed and 29 patients without the placement of the external fixation. Their baseline characteristics and functional outcomes were evaluated and compared.

**Results:**

The average follow-up time was 28.4 ± 3.7 months. There were no significant differences in the ROM of the elbow, MEPS, VAS, DASH, or complications between the two groups. The operation time and treatment cost of the patients without external fixation were significantly lower than patients with external fixation.

**Conclusion:**

Combined medial-lateral approach open elbow arthrolysis without external fixation is a safe and effective way to treat elbow stiffness.

**Level of evidence:**

Therapeutic Level III; Retrospective Cohort Comparison; Treatment Study.

## Background

Elbow stiffness is defined as the compromised elbow function and motion due to traumatic or non-traumatic causes. Evidence showed that 12 % of patients developed elbow stiffness after elbow injuries [1–3]. Morrey et al. [4] stated that the elbow functional range of motion (ROM) exceeding 30° - 130° of extension-flexion and 50° of pronation-supination is sufficient for daily activities. A loss of 50° in the flexion-extension arc has been reported to cause an 80% loss of function [4]. Nowadays, due to the wide application of keyboards, mice and mobile phones, the ROM required by the elbow is larger than before [5]. Therefore, it is generally believed that the elbow stiffness can be diagnosed when the patient’s elbow fails to accomplish daily activities due to the reduced ROM. Elbow stiffness can be divided into four degrees according to the extension-flexion arc: ≤30° is extremely severe, 31° ~ 60° is severe, 61° ~ 90° is moderate, and > 90° is mild [6].

Open arthrolysis is the most commonly used surgical method for elbow stiffness. For severe elbow stiffness (extension-flexion arc ≤60°), it is very difficult to achieve a complete restoration of ROM while preserving the stability of the elbow through the arthrolysis. Therefore, surgeons usually use hinged external fixation for several weeks to maintain the stability of the elbow after open arthrolysis. However, the application of hinged external fixation has many complications, including pin site infection, pin breakage, and radial nerve injury. It also lowers the postoperative quality of life and incurs high hospitalization expense [7–10]. Since 2018, our institution started to treat patients with severe elbow using the combined medial-lateral approach arthrolysis. This study aimed to compare the functional outcomes of the arthrolysis with and without hinged external fixation.

## Methods

### Patients

We retrospectively collected the data of patients with severe elbow stiffness at our institution between January 2018 and January 2019. We obtained institutional review board approval for this retrospective investigation, and informed consent was obtained from each patient. The inclusion criteria were (1) 18 to 60 years old; (2) severe elbow stiffness (extension-flexion arc ≤60°); and (3) treated by combined medial-lateral approach arthrolysis. The exclusion criteria were (1) concomitant burn or central nervous system injury; (2) elbows with rheumatoid arthritis or bone tumors; (3) follow-up time less than 2 years; or (4) incomplete clinical data.

**Fig. 1:**
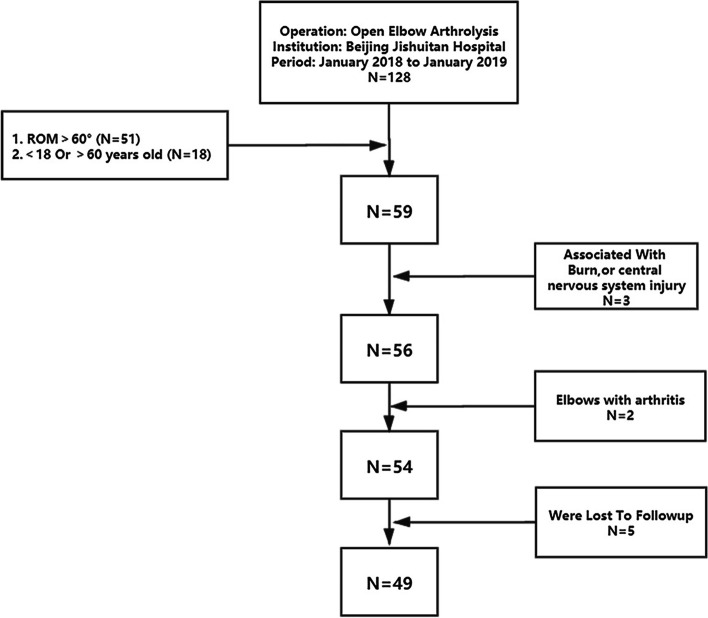
The flowchart shows the inclusion and exclusion criteria

A total of 128 patients underwent arthrolysis at our institution. After applying the inclusion and exclusion criteria, 49 patients were included in the study (Fig. 1), with a mean follow-up time of 28.4 ± 3.7 months (24–35 months). We measured the ROM of the elbow [11]. To objectively evaluate the elbow function, the Mayo Elbow Performance Score (MEPS) was used with regard to 4 aspects: pain (45 points), ROM (20 points), stability (10 points), and the ability to accomplish daily activities (25 points) [12]. The Disabilities of the Arm, Shoulder and Hand (DASH) questionnaire was used to subjectively evaluate elbow-related symptoms and disability [13]. The decision of whether using the hinged external fixation was made according to the preferences of the surgeons and the patients’ consent. The patients were then categorized into two groups: with hinged external fixation (group A) or without hinged external fixation (group B). All surgeries were performed by the same two surgeons, who were both well-trained and experienced in elbow arthrolysis. Group A consisted of 20 patients (16 males and 4 females), with an average age of 34.1 ± 10.8. Group B consisted of 29 patients (19 males and 10 females), with an average age of 37.6 ± 11.2 years. There were no significant differences in average age, sex, surgical side, body mass index (BMI) or incision type between the two groups (Table 1, *P*>0.05). The original types of injury of patients in the two groups are shown in Table 1.

**Table 1:** Comparison of the baseline characteristics between the two groups

		Group A (20)	Group B (29)	*P* Value
Age (Mean ± SD)		34.1 ± 10.8	37.6 ± 11.2	0.272
Sex (male/female)		16/4	19/10	0.344
Side (left/right)		12/8	11/18	0.155
BMI		24.9 ± 4.3	24.8 ± 4.6	0.906
Incision (posterior midline/ lateral and medial)		17/12	12/8	1.000
The original injury types of patients	Radial head fracture	3	7	0.564
	Olecranon fracture	5	10	
	Distal humeral fracture	7	8	
	Terrible triad of elbow	2	2	
	Coronoid process fracture	2	1	
	Elbow dislocation	0	1	
	Prolonged immobilization	2	0	

### Surgical technique

Patients were placed in the standard supine position under general anesthesia or brachial plexus block. Sterile air tourniquets were applied.

A posterior midline incision or lateral and medial incisions were selected according to the previous surgical scar, location of heterotopic ossification (HO), and cause of stiffness. There was no significant difference in the choice of surgical incision between the two groups. (P>0.05, Table 1).

**Fig. 2:**
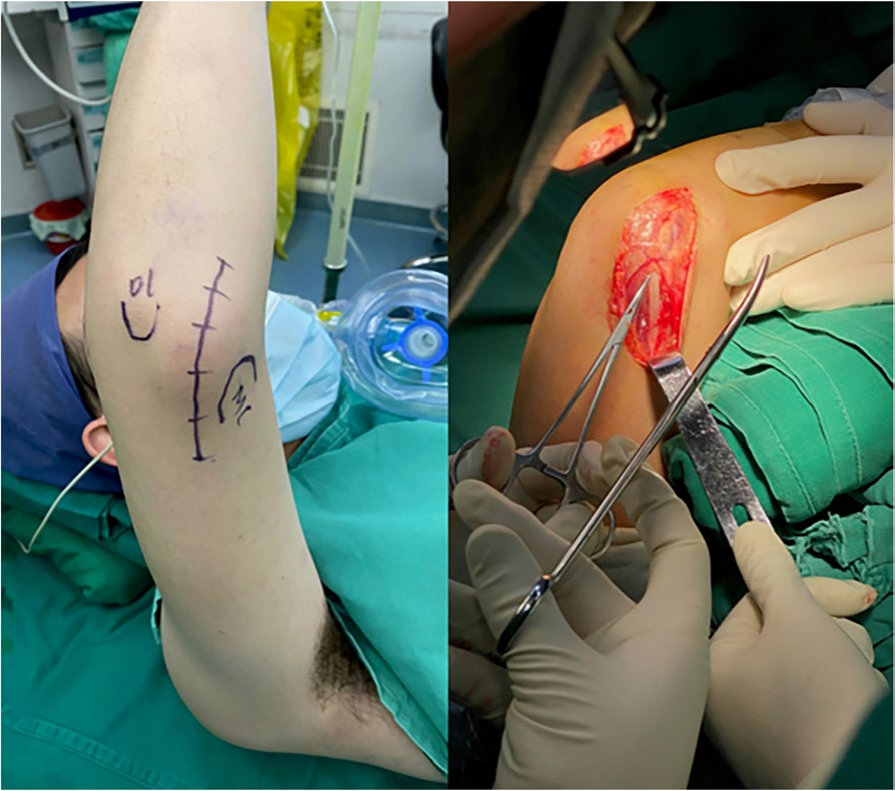
The medial incision of combined medial-lateral approach arthrolysis

For the combined medial-lateral approach, the elbow was accessed medially first (Fig. 2). The ulnar nerve was exposed and protected. The triceps was reflected to the lateral side to exposed the medial and posterior aspect of the joint, and then the surgeons excised the HO on the posteromedial side of the joint, the posterior capsule and transverse and posterior bundle of the medial collateral ligament (MCL) while the anterior bundle was preserved. If there was still blockage posteriorly, the surgeons would perform olecranon fossa osteoplasty and olecranon tip excision in a consecutive manner.

**Fig. 3:**
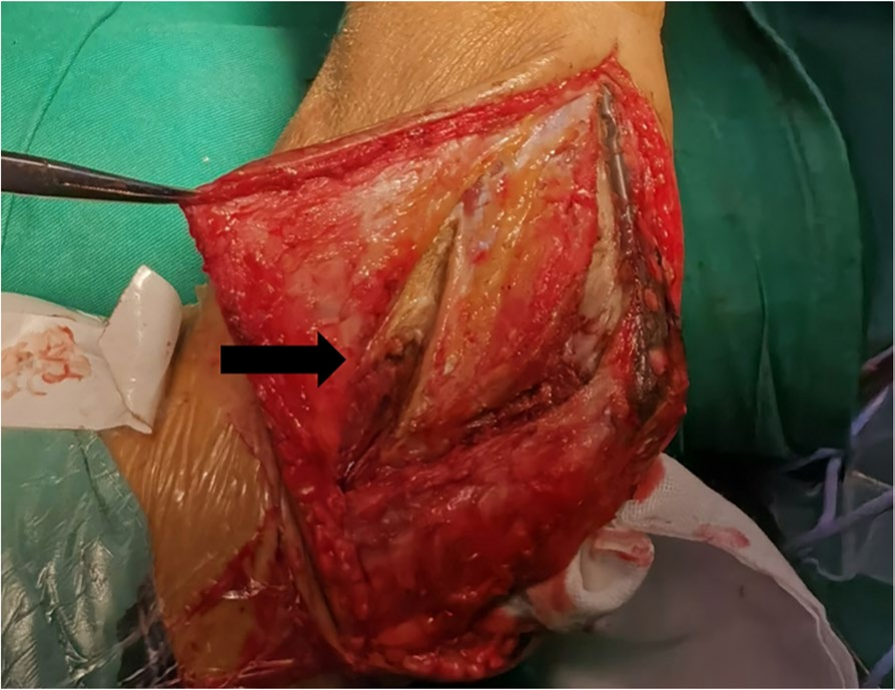
The lateral column procedure with partial EDC split

On the lateral side, the lateral column procedure was done with partial extensor digitorum communis (EDC) split (Fig. 3) to expose the anterolateral part of the joint while preserving the lateral ulnar collateral ligament (LUCL). Surgeons then performed radial head fossa, coronoid fossa and coronoid process osteoplasty, and released the anterior capsule. If there was bony blockage on the posterolateral side, the surgeons would incise along the lateral margin of the triceps to expose the lateral ulnohumeral joint, excise the HO and release the posterolateral joint capsule.

**Fig. 4:**
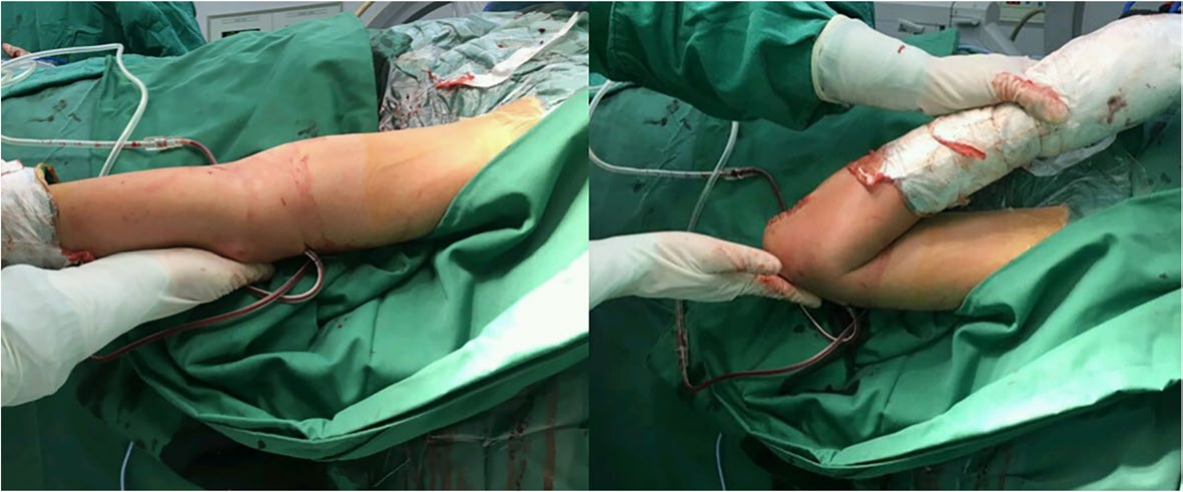
The elbow was extended and flexed passively under direct vision after arthrolysis

The surgery was only considered complete when a satisfactory passive (moderate force) ROM was achieved under direct vision (0° - 130° of extension-flexion. Figure 4). Lateral stress test and lateral pivot shift test were performed to assess elbow stability [14]. Then, the ulnar nerve was transposed anteriorly to reduce post-operative ulnar nerve palsy. The fascia and subcutaneous tissue were closed layer by layer, and the incision was closed after the placement of drainage. Finally, a Stryker DJD II hinged external fixator was applied to the elbow based on the surgeon’s preference and patient’s consent.

### Postoperative treatment

All patients received analgesia pumps for 3 days after surgery to alleviate postoperative pain and help postoperative exercise. Closed negative pressure drainage was left in place until the 24-h drainage volume was less than 30 ml. The patients received oral indomethacin 25 mg/time, 3 times/day, for 6 weeks. On the second post-operative day, patients began to exercises by continuously flexing and extensing elbow and pronating and supinating the forearm with the assistance of the other arm. The external fixation was removed 6–8 weeks after surgery.

**Fig. 5:**
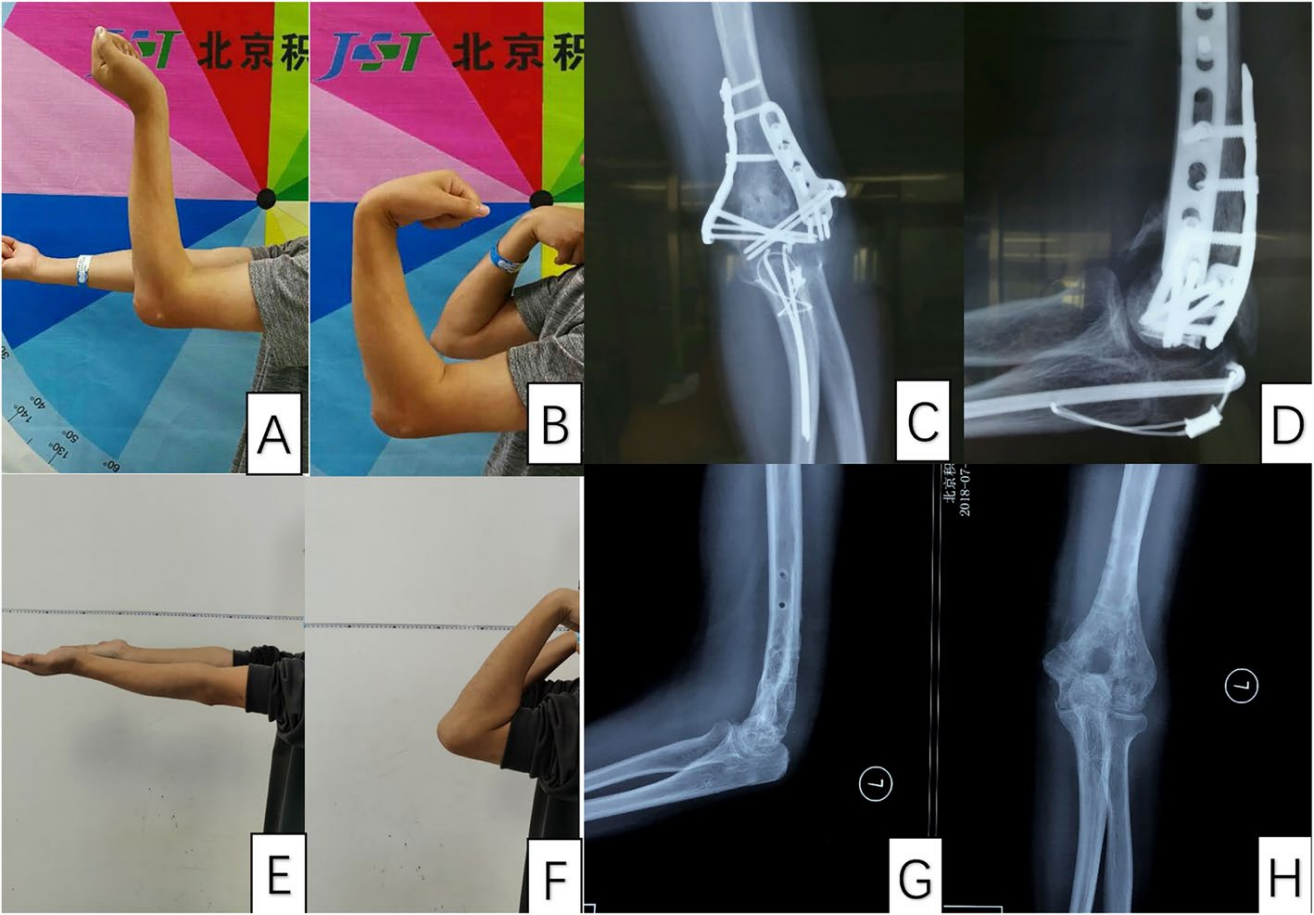
Elbow function and X-ray preoperatively and postoperatively. (A and B) Preoperative extension and flexion. (C and D) Preoperative X-rays. (E and F) Extension and flexion at the last follow-up. (G and H) The X-rays at the last follow-up

Patients were routinely followed up at 1, 3, 6 and 12 months postoperatively, and the last follow-up was performed for all patients 2 year after the operation. We measured the ROM of the elbow, MEPS, DASH, visual analogue scale (VAS) and documented the complications. Postoperative standard anteroposterior and lateral X-rays of the elbows were taken, and examined to find the signs of joint degeneration and the evidence of newly formed HO (Fig. 5).

### Statistical analysis

Statistical analyses were performed using SPSS software for Windows (IBM SPSS Statistics, version 24; IBM, Armonk, NY, USA). The changes in the ROM, VAS, MEPS and DASH in the same group were tested by paired t-tests. Then, the independent sample t-test was used to compare the perioperative data and improvement of functional outcome of patients between the two groups. The chi-square test was performed to compare the incidences of complications between groups. *P* < 0.05 was considered statistically significant.

## Results

Firstly, the ROM of patients in the two groups were compared before surgery and at the last follow-up (Table 2). The ROM and MEPS of all patients increased significantly after the surgery and rehabilitation compared to the pre-operative state (*P* < 0.05) while VAS and DASH scores decreased significantly (*P* < 0.05), indicating improvement of elbow function.

**Table 2:** Comparison of the functional outcomes between pre-operation and the last follow-up

		ROM of flexion and extension	ROM of rotation	VAS	MEPS	DASH
Group A	pre-operation	25.8 ± 23.0	106.5 ± 52.8	1.7 ± 1.9	58.3 ± 15.7	53.7 ± 16.6
	the last follow-up	132.1 ± 15.8	143.6 ± 22.2	0.3 ± 0.5	97.0 ± 5.5	6.5 ± 4.8
	P	0.000	0.001	0.007	0.000	0.000
Group B	pre-operation	26.7 ± 21.8	121.9 ± 49.7	2.6 ± 2.1	51.4 ± 11.3	46.7 ± 15.0
	the last follow-up	133.5 ± 16.1	140.1 ± 21.2	0.2 ± 0.6	96.4 ± 6.1	6.1 ± 4.0
	P	0.000	0.040	0.000	0.000	0.000

Subsequently, we made comparison of the perioperative data between the two groups (Table 3). There was no significant difference in hospitalization time (*P* = 0.069). The length of operation time and cost of treatment of group A were greater than those of group B (P<0.05).

**Table 3:** Comparison of the functional outcomes between the two groups

	Group A (20)	Group B (29)	*P* value
Hospitalization time (days)	11.6 ± 3.9	9.9 ± 2.3	0.069
Operation time (minutes)	155.1 ± 60.7	106.9 ± 46.4	0.003
Intraoperative blood loss (ml)	151.0 ± 80.6	89.3 ± 55.7	0.003
Treatment cost (K yuan)	54.7 ± 15.9	41.7 ± 13.3	0.003

Then, we compared the degree of improvement in the functional outcome of patients between the two groups (Table 4). No significant difference was found between the two groups concerning the improvement of ROM, VAS, MEPS, or DASH (Table 4, *P* > 0.05). In group A, ulnar nerve symptoms were observed in 11 patients (55%) post-operatively and 8 of which had completely recovered at the last follow-up. Elbow pain was observed in 4 patients (20%) and all had relieved at the last follow-up. One patient (5%) had recurrence of HO at the last follow-up. In group B, 15 patients (51.7%) had ulnar nerve symptoms, and 13 patients had completely recovered at the last follow-up. Five patients (17.2%) had elbow pain, all of whom were relieved at the last follow-up. Two patients (6.5%) had recurrence of HO at the last follow-up. The recurrence of HO didn’t affect the elbow functional outcomes of the three patients. There was no patient presented with surgical site infection or elbow instability. No significant difference concerning the rates of ulnar nerve symptoms (*P* = 1.000), pain (*P* = 0.320) and recurrence of HO (*P* = 1.000) between the two groups was noticed (Table 5). In addition, there were 3 cases of pin site infection in group A before the removal of the external fixation and all of the pin tracts healed spontaneously after the removal of the pins.

**Table 4:** Comparison of the degree of improvement in the functional outcomes between the two groups

		Group A (20)	Group B (29)	*P* value
Functional prognostic change	ROM of flexion and extension	106.3 ± 27.2	106.8 ± 27.0	0.942
	ROM of rotation	37.1 ± 40.1	18.2 ± 45.5	0.140
	VAS	−1.4 ± 2.0	−2.3 ± 2.1	0.106
	MEPS	38.8 ± 17.3	45.0 ± 10.9	0.128
	DASH	− 47.2 ± 17.1	−40.6 ± 13.8	0.146

**Table 5:** Comparison of the complications between the two groups

		Group A (20)	Group B (29)	*P* value
Compli-cations	Ulnar nerve symptoms (finally)	3 (15%)	2 (6.9%)	1.000
	Elbow pain	4 (20%)	5 (17.2%)	0.320
	Recurrence of HO	1 (5%)	2 (6.9%)	1.000
	Pin site infection	3	–	–

## Discussion

Open elbow arthrolysis is an effective treatment modality for elbow stiffness [15]. The restoration of the elbow function is mainly achieved through the excision of bony blockage, contracted joint capsule and scar tissue [16, 17]. In the past, several studies reported the use of open arthrolysis in the treatment of elbow stiffness and provided the evidence that open arthrolysis could effectively improve elbow function and the quality of patients’ life [18–22].

The important primary stabilizers of the elbow include the anterior bundle of the medial collateral ligament (aMCL), the LUCL (an important part of the lateral collateral ligament complex), and the congruence of the ulnohumeral joint [23–31].

In severe elbow stiffness, the soft tissue contracture and HO often contribute to the compromised elbow function. During the elbow release procedure, the aMCL and LUCL are often partially or completely damaged, resulting in instability of the elbow [32–34].

In order to avoid instability after arthrolysis, some surgeons use the technique of hinged external fixation. Zhou et al. [35] reported the functional outcome of 38 cases of open arthrolysis combined with external fixation (31 months of follow-up). The average ROM of flexion and extension arc increased from 27° to 126°, and the ROM of rotation increased from 148° to 153°. Sun et al. [36] reported the five-year follow-up results of 49 cases of open arthrolysis in combination with external fixation. The flexion-extension ROM increased from 27 degrees to 131°, and the average MEPS increased from 54 to 95; Kulkarni et al. [37] retrospectively analyzed 26 cases of open relaxation combined with external fixation (5.2 years of follow-up). The flexion-extension range improved by 102.4°, and the average MEPS increased by 44. In all these studies, the hinged external fixation provided stability for the elbow immediately after the surgery, which allowed safe and early exercise [38–40].

However, several studies also reported external fixation related complications such as pin tract infection, pin breakage, and radial nerve injury [36], as well as higher hospitalization costs and longer operation times [7–10]. Ring et al. [41] retrospectively compared the prognosis of patients with elbow stiffness who underwent elbow release with external fixation (23 cases) or without external fixation (19 cases). The changes in ROM of the two groups showed no statistical significant difference (89° VS 78°, P>0.05). In the external fixation group, 5 patients developed pin loosening and infection, 1 patient had ulnar fracture, and 2 patients had pin breakage.

For patients with severe stiffness, completely removing the HO and soft-tissue contracture of the elbow while preserving the aMCL and LUCL is still a fair challenge. To solve this problem, our institution modified the traditional elbow arthrolysis procedure. In previous studies, the anterior part of the joint were often released through the medial over-the-top approach [22, 42], which inevitably damages the aMCL. On the lateral side, the anterolateral joint was accessed through extended Kocher approach [19, 43, 44], which would often damage the LUCL. The damage to the aMCL or LUCL would compromise the stability of the elbow. In our study, we used the combined medial-lateral approach. Our procedure on the medial side focused on the release of the posterior joint capsule, while protecting the aMCL. On the lateral side, the lateral column procedure was performed with partial EDC split instead of Kocher approach. Therefore, the LUCL always remains intact. After the excision of HO and soft-tissue contracture, the lateral stress test and lateral pivot shift test were performed to evaluate the elbow stability, and none of the patients in our study showed immediate elbow instability.

The decision of whether using the hinged external fixation was made according to the preferences of the surgeons and the patients’ consent. Although the stability of the elbow was confirmed intra-operatively, some surgeons still preferred to use an external fixator after arthrolysis in order to avoid instability of the elbow during early stage rehabilitation. While other surgeons thought the use of external fixation was not necessary for early rehabilitation. In addition, surgeons would tell the patients the pros and cons about the external fixation before operation, and some patients refused to accept the use of it because of the inconvenience and the increased treatment cost. This study compared the functional results of the two groups of patients with or without external fixation after combined medial-lateral approach arthrolysis in our hospital. Compared with preoperative data, the flexion and extension ROM, rotational ROM, MEPS, VAS, and DASH were significantly improved (P<0.05), suggesting that both methods could achieve satisfactory functional outcomes of these patients.

Furthermore, we compared the functional result between patients with and without the external fixation. There were no statistically significant difference in the ROM, MEPS, DASH, VAS (P>0.05). Additionally, the operation time and the cost of the patients without external fixation were significantly lower than those with external fixation. Pin tract infection was found in three patients who received the external fixation. These results confirmed that for patients with severe elbow stiffness, the use of combined medial-lateral approach arthrolysis without external fixation could improve the functional outcomes of elbows and was relatively safe.

This study has the following limitations: (1) as a retrospective study, the result is prone to have selection bias; (2) the size of this study, though comparable to or even larger than similar studies, may not be large enough to show the difference between groups; (3) the measurement of ROM of elbows was performed by the same doctor, and there may have been favour detection bias; and (4) the surgeries were performed by two surgeons, and there may have been bias to the surgical outcomes.

## Conclusion

The use of combined medial-lateral approach open elbow arthrolysis to treat severe elbow stiffness significantly improves the ROM and function of the elbow. The stability of the elbows can be achieved without external fixation. Not using the external fixation spares patients the complications and inconvenience of external fixation. Furthermore, the use of external fixation lengthens hospitalization cost and increases the operation time.

## Data Availability

The datasets generated and/or analysed during the current study are not publicly available due to limitations of ethical approval involving the patient data and anonymity but are available from the corresponding author on reasonable request.

## Abbreviations

ROM: Range of motion
MEPS: The Mayo Elbow Performance Score
DASH: The Disabilities of the Arm, Shoulder and Hand
BMI: Body mass index
HO: Heterotopic ossification
MCL: The medial collateral ligament
EDC: Extensor digitorum communis
LUCL: The lateral ulnar collateral ligament
VAS: Visual analogue scale
aMCL: The anterior bundle of the medial collateral ligament
